# 6-Hydroxydopamine Induces Neurodegeneration in Terminally Differentiated SH-SY5Y Neuroblastoma Cells via Enrichment of the Nucleosomal Degradation Pathway: a Global Proteomics Approach

**DOI:** 10.1007/s12031-021-01962-z

**Published:** 2022-03-08

**Authors:** Kasthuri Bai Magalingam, Sushela Devi Somanath, Premdass Ramdas, Nagaraja Haleagrahara, Ammu Kutty Radhakrishnan

**Affiliations:** 1 School of Postgraduate Studies, International Medical University, Kuala Lumpur, Malaysia; 2 Jeffrey Cheah School of Medicine and Health Sciences, Monash University, Malaysia, Bandar Sunway, Malaysia; 3 Pathology Division, School of Medicine, International Medical University, Kuala Lumpur, Malaysia; 4 Division of Applied Biomedical Sciences and Biotechnology, School of Health Sciences, International Medical University, Kuala Lumpur, Malaysia; 5 College of Public Health, Medicine and Veterinary Sciences, James Cook University, Townsville, QLD 4811 Australia; 6 Monash-Industry Palm Oil Education and Research Platform (MIPO), Monash University Malaysia, Bandar Sunway, Selangor, Malaysia

**Keywords:** Parkinson’s disease (PD), 6-Hydroxydopamine (6-OHDA), Human neuroblastoma cells (SH-SY5Y)

## Abstract

The SH-SY5Y human neuroblastoma cells have been used for decades as a cell-based model of dopaminergic neurons to explore the underlying science of cellular and molecular mechanisms of neurodegeneration in Parkinson’s disease (PD). However, data revealing the protein expression changes in 6-OHDA induced cytotoxicity in differentiated SH-SY5Y cells remain void. Therefore, we investigated the differentially regulated proteins expressed in terminally differentiated SH-SY5Y cells (differ-SH-SY5Y neural cells) exposed to 6-hydroxydopamine (6-OHDA) using the LC–MS/MS technology and construed the data using the online bioinformatics databases such as PANTHER, STRING, and KEGG. Our studies demonstrated that the neuronal development in differ-SH-SY5Y neural cells was indicated by the overexpression of proteins responsible for neurite formations such as calnexin (CANX) and calreticulin (CALR) besides significant downregulation of ribosomal proteins. The enrichment of the KEGG ribosome pathway was detected with significant downregulation (*p* < 0.05) of all the 21 ribosomal proteins in differ-SH-SY5Y neural cells compared with undifferentiated cells. Whereas in the PD model, the pathological changes induced by 6-OHDA were indicated by the presence of unfolded and misfolded proteins, which triggered the response of 10 kDa heat shock proteins (HSP), namely HSPE1 and HSPA9. Moreover, the 6-OHDA-induced neurodegeneration in differ-SH-SY5Y neural cells also upregulated the voltage-dependent anion-selective channel protein 1 (VDAC1) protein and enriched the KEGG systemic lupus erythematosus (SLE) pathway that was regulated by 17 histone proteins (*p* < 0.05) in differ-SH-SY5Y neural cells. These results suggest that the nucleosomal degradation pathway may have regulated the 6-OHDA induced neurodegeneration in PD cell-based model, which is reflected by increased apoptosis and histone release in differ-SH-SY5Y neural cells.

**Supplementary Information:**

The online version contains supplementary material available at 10.1007/s12031-021-01962-z.

## Introduction

Over the last 20 years, a significant amount of data has emerged suggesting different cellular models that are valuable in evaluating potential drug targets in modulating the disease course in PD. Undeniably, the immortalized SH-SY5Y human neuroblastoma cell line has been an excellent choice for the preliminary drug screening for PD. The SH-SY5Y cell line demonstrates a moderate activity of crucial neuronal markers that usually exists in dopaminergic cells such as dopamine-β-hydroxylase (Katsuyama et al*.* 2021), tyrosine hydroxylase (Khwanraj et al*.* 2015), choline acetyl-transferase (Filograna et al*.* 2015) and noradrenaline (Kovalevich and Langford 2013). Whether undifferentiated or differentiated SH-SY5Y neuroblastoma should be used as a substitute to dopaminergic neurons in PD-related studies remain controversial. However, most neuroscience researchers have suggested that it is of great importance to utilize the differentiated SH-SY5Y human neuroblastoma cells that resemble human dopamine neurons for PD studies (Magalingam et al. 2020).

A fantastic wealth of information has been accumulated on different techniques and protocols to develop terminally matured dopamine neurons. Cheung et al*.* have pointed out that retinoic acid (RA) induced differentiation of SH-SY5Y cells demonstrated extensive outgrowth of neurite and augmentation of neuronal markers such as neuronal nuclei, enolase, synaptophysin and synaptic-associated protein-97 (Cheung et al*.* 2009). Studies have also shown that RA-induced differentiation exhibits low proliferative rate and increased expression of genes related to synaptic vesicle cycle, dopamine synthesis/ degradation and dopamine transporter (DAT) (Lopes et al. 2017), as well as higher tyrosine hydroxylase, which is the critical enzyme in the synthesis of dopamine neurotransmitter (Khwanraj et al*.* 2015). Furthermore, our previous studies have delineated that the RA-induced differentiation on SH-SY5Y cells in a low-serum culture medium demonstrated enhanced neurite projection with longer varicosities connecting the adjacent cells, reduced proliferation rate as well as increased levels of dopamine and alpha-synuclein. Moreover, the differentiated neural cells also expresses dopaminergic characteristics at the genetic level as evidenced in upregulation of the DRD2 fold change expression (Magalingam et al. 2020). Our findings correlate with numerous previous studies that suggested that differentiated SH-SY5Y cell line possesses the closest resemblance with dopaminergic cells and is suitable for neurodegenerative related studies (Khwanraj et al*.* 2015; Lopes et al. 2017).

Although there is ample evidence on various approaches to establishing differentiated neuronal cells, data on the 6-OHDA induced cytotoxicity on differentiated SH-SY5Y cells remains scarce. Lopes et al*.* (2017) and Cheung et al*.* (2009) have shown that 6-OHDA have varied susceptibility on RA-differentiated SH-SY5Y cells. Other studies have documented the biochemical changes induced by 6-OHDA on undifferentiated SH-SY5Y cells. These studies have shown that 6-OHDA-induced cytotoxicity is mediated by mitochondrial fragmentation (Gomez-Lazaro et al*.* 2008), autophagy (Arsikin et al*.* 2012), mitochondrial respiration (Iglesias-González et al*.* 2012), generation of free radicals (Storch et al*.* 2000) and changes in lipid classes (Xicoy et al*.* 2020). Since there is no data available on the proteomic changes implicated by 6-OHDA on differentiated SH-SY5Y cells, our findings contribute to filling the knowledge gap on the molecular, biological and pathways regulated by the 6-OHDA on RA-differentiated SH-SY5Y neuroblastoma cells.

The proteomic study is an emerging field of large-scale protein expression study that provides an unprecedented insight into the cellular structural and functional framework. Intrinsically, proteins within the cell play a crucial role in providing cellular structure, movement and communication, and participate in metabolism, respiration, signal transduction and reproduction activities (Aslam et al*.* 2017). The differential expression of proteomes in cells is a signal of significant alterations in cellular activity. The label-free liquid chromatography coupled to tandem mass spectrometry (LC–MS/MS) technology quantified and revealed thousands of global proteins across samples from digested peptides and matched the detected peptide/protein identity using automated database searching (Sinitcyn et al. 2018). Subsequently, the Protein ANalysis THrough Evolutionary Relationships (PANTHER) (Mi et al*.* 2019) bioinformatic database was applied to analyse the differentially regulated proteins for their functional annotation using gene ontology (GO) (Pomaznoy et al. 2018). The Search Tool for the Retrieval of Interacting Genes/Proteins (STRING) (Szklarczyk et al*.* 2019) and Kyoto Encyclopaedia of Genes and Genomes (KEGG) (Kanehisa 2019) search engines were utilized to understand the protein–protein interaction, enriched bio-pathway and molecular function.

In the present study, we performed label-free global protein profiling of terminally differentiated SH-SY5Y (differ-SH-SY5Y) neural cells and the cell model of PD using 6-hydroxydopamine-induced neurodegeneration of differ-SH-SY5Y cells based on a shotgun proteomic methodology. The main objective of this study is to identify the novel biomarkers and pathways involved in the neuronal development of SH-SY5Y neuroblastoma cells and cellular model of PD. The functional role and enriched canonical pathway of the differentially expressed proteins identified from these studies were further explored using bioinformatic tools. This study further authenticates the use of the 6-OHDA induced cytotoxicity on differ-SH-SY5Y cell model as an important tool in the investigation of potential drug targets for PD.

## Methods

### Cell Culture

The SH-SY5Y neuroblastoma cells (Cat # CRL-2266) were grown in a complete culture medium containing 88% of Dulbecco Modified Eagle Medium (DMEM) supplemented with 4.5 g/L glucose and L-glutamine without sodium pyruvate (Corning, Corning, NY, U.S.A), 10% heat-inactivated fetal bovine serum (FBS, Biosera, Nuaille, France), 1% Penicillin–streptomycin (P/S) (Gibco, Carlsbad, USA) and 1% non-essential amino acid (NEAA, GIBCO, Carlsbad, USA) 37 °C in a humidified 5% CO_2_ incubator. The culture medium was replaced every 3-days, and cells were sub-cultured once it reaches 70% confluence.

### Differentiation of SH-SY5Y Neuroblastoma Cells

The establishment of differ-SH-SY5Y neural cells was performed by seeding 1 × 10^5^ cells/mL of SH-SY5Y human neuroblastoma cells in a complete culture medium in a T75 flask. After 24 h of cell seeding, the cells were exposed to a differentiation medium containing 95% of DMEM, 3% heat-activated FBS, 1% P/S, 1% NEAA and 10 μM of all trans-retinoic acid (RA, Sigma Aldrich, St. Louis, USA) for 6-days. After 6-days of the differentiation phase, the differ-SH-SY5Y neural cells express dopaminergic characteristics at morphological, biochemical and genetic levels (Magalingam et al. 2020).

### Preparation and Treatment Protocol of 6-Hydroxydopamine

6-Hydroxydopamine (6-OHDA) (Sigma Aldrich, St. Louis, USA) was freshly prepared using chilled 0.15% ascorbic acid (Sigma Aldrich, St. Louis, USA) and sterilized by filtering through a syringe filter fitted with a 0.2-μM filter and stored in the dark at 4°C to protect it from light. On day 7 (after the 6-days of differentiation phase), the differ-SH-SY5Y cells were exposed to 10 μg/mL of 6-OHDA in a serum-free culture medium for 24 h. The undifferentiated cells were maintained in a serum-free culture medium without 6-OHDA for the same duration of time. The concentration of 6-OHDA (10 μg/mL) was selected based on the preliminary studies that displayed inhibition of differ-SH-SY5Y neural cell proliferation by about 50%.

### Protein Extraction

On day 8, the undifferentiated, differ-SH-SY5Y, and 6-OHDA exposed differ-SH-SY5Y neural cells were harvested from the T75 flasks and recovered by centrifugation *(1000 g for 10 min at 4 °C)*. The supernatant was discarded, and the total protein was extracted from the cells using the EasyPrep Mini MS Sample Prep kit (Thermo Fisher Scientific, USA). Briefly, 100 µL of lysis buffer *(provided with the kit)* and 1 µL of universal nuclease *(provided with the kit)* were added to the cell pellets and thoroughly mixed until the sample's viscosity reduced. The extracted protein samples were aliquoted and stored at -80 °C until further use.

### Determination of Protein Concentration

The extracted proteins’ concentration was estimated using the Pierce BCA Protein Kit (Thermo Fisher Scientific, USA) (El-Rami et al. 2017). The absorbance was measured at 562 nm using a microplate reader (SpectramaxM, CA, USA). A standard curve was prepared by plotting the average corrected absorbance measurements of BSA standards vs concentration in µg/mL to determine the ‘unknown samples’ protein concentrations.

### Protein Reduction and Alkylation

The extracted proteins were subjected to reduction and alkylation steps followed by protein digestion and a “clean-up” procedure using the EasyPrep Mini MS Sample Prep kit protocol. The samples were removed from the -80 °C freezer and thawed at room temperature. Once samples were liquefied, 100 µg of protein from each sample was transferred into appropriately labelled sterile 1.5-mL tubes, and the final volume for each sample was adjusted to 100 µL with lysis solution (provided with the kit). Then, 50 µL of reduction solution (provided with the kit) was added to each tube and mixed gently. Following this, 50 µL of alkylation solution (provided with the kit) was added to the tubes, and the tubes were gently mixed. The tubes were incubated at 95 °C using a heat block for 10 min to reduce and alkylation reactions. Following this, the samples were removed from the heat block to cool to room temperature and subjected to Trypsin/Lys-C protein digestion procedure.

### Trypsin/Lys-C Protein Digestion

For the digestion step, 500 µL of the enzyme reconstitution solution (provided with the kit) was added to a vial containing Trypsin/Lys-C-Protease mix (provided with the kit). Then, 50 μL of the reconstituted Trypsin/Lys-C-Protease mix was added to each tube containing the samples, and the tubes were incubated at 37 °C with shaking for 3 h to allow protein digestion to take place. At the end of 3 h, 50 µL of digestion stop solution (provided with the kit) was added to each tube to terminate the digestion process.

### Peptide Clean-up

After the peptide digestion, any contaminants present in the samples were sequentially removed using the peptide clean-up column (provided with the kit)*.* The peptide clean-up columns were labelled accordingly to avoid any sample mix-ups. As per the manufacturer recommended protocol, the white cap at the bottom of each peptide clean-up column was removed, and its green top cap loosened before each clean-up column was placed in individual 2 mL tubes. The tubes were centrifuged *(3000 g for 2 min)* to remove trapped liquid from the column, and the flow-through from each column was discarded. The digested peptides from each sample were transferred into the respective peptide clean-up column. The columns were centrifuged *(1500 g for 2 min)*, and the flow-through from each column was discarded. Next, wash solution A *(provided with the kit)* was added into each column before centrifugation, and the flow-through from each column was discarded. This step was repeated using wash solution B *(provided with the kit)*. Before the elution step, the peptide columns were centrifuged to remove any residual fluid. Then, each column was placed on appropriately labelled sterile collection tubes, and elution solution *(provided with the kit)* was added to each column. The eluted peptide samples were collected by centrifugation, dried using a vacuum centrifuge and stored at -80 °C before LC–MS/MS analysis.

### Liquid Chromatography and Mass Spectrometry Analysis

The digested peptides were loaded into an Agilent 1200 HPLC-Chip/MS Interface, coupled with Agilent 6550 iFunnel Q-TOF LC/MS (Agilent, Santa Clara, CA, USA). The column was equilibrated with 0.1% formic acid in water (solution A). The peptides were eluted from the column with 90% acetonitrile in 0.1% formic acid in water (solution B). Quadrupole-time of flight (Q-TOF) polarity was set at positive with capillary and fragmenter voltage being set at 1900 V and 360 V, respectively, and 5 L/min of gas flow with a temperature of 325 ◦C. The collision energy was determined at 3.7 V (100 Da), and reference masses with positive polarity was set at 299.294457 and 1221.990637. The peptide spectrum was analysed in auto MS mode ranging from 110–3000 m/z for MS scan and 50–3000 m/z for MS/MS scan.

### Data Computation

The raw data of tryptic peptides were extracted and processed using PEAKS X software (Bioinformatics Solutions Inc., Waterloo, ON, Canada) using Uniprot, Swissprot and TrEMBL databases. The PEAKS X software allows for the determination of the protein abundance using the following search parameters: retention time lower bound: ≥ 0, retention time upper bound: ≤ 55, average area: ≥ 0, charge lower bound: ≥ 1, confident number samples per group: ≥ 1, peptide identification count: ≥ 1, protein significance: ≥ 20, used peptides: ≥ 1, fixed modification: Carbamidomethylation of cysteine residues and false discovery rate (FDR): 1% in three biological replicate injections. Protein abundance was computed using normalized spectral protein intensity (LFQ intensity). The obtained peptide/protein list was exported to Microsoft Excel to quantitate three biological replicates from the same samples were grouped in the same matrix. The protein data were filtered for at least two valid values, and protein only presented in one biological replicate was eliminated. The biological replicates from all samples were clustered under the same matrix, and the missing data were assigned with a random number derived from a normal distribution. The reason for the missing data measurement is due to low protein abundance in LC–MS/MS analysis.

### Biocomputational Protein analysis

## Protein Functional Classification 

The identified proteins with *p* < 0.05 against their respective controls were classified based on (i) molecular function, (ii) biological process and (iii) cellular component using Gene Ontology (GO) term analysis. The online bioinformatics tool PANTHER database (http://pantherdb.org) version 16 was used to elucidate the functions of these differentially expressed proteins in the differ-SH-SY5Y neural cells and 6-OHDA exposed differ-SH-SY5Y cells. Only the top 10 enriched GO terms were listed for each functional classification. All results displayed expressed adjusted *p-value* < 0.05 as determined by Fischer's Exact test and FDR.

## Protein–Protein Interaction (PPI) Analysis

The STRING database (STRINGv11.0) (https://string-db.org) was used to construct the PPI network in neuronal maturation and oxidative stress in 6-OHDA treated neural cells based on its physical binding and regulatory interaction. The Uniprot IDs of the differentially regulated proteins were inputted in the STRING database under the multiple protein analysis categories and followed by the selection of *Homo Sapiens* from organism pull-down selection. The basic settings that were used in the analysis of PPI are Network type: Full network, active interaction sources: text mining, neighbourhood, experiments, databases, co-expression, gene-fusion, co-occurrence, Minimum required interaction score: highest confidence (0.9) and K-mean clustering was specified as three clusters. Thick edges between the protein nodes demonstrate strong protein interaction. This protein cluster was uploaded into Cytoscape 3.8.0 to visualise the complex networks by integrating Log2 fold-change data.

## Pathway Enrichment Analysis

The pathway enrichment analysis of the differentially expressed proteins was performed using DAVID (Database for Annotation, Visualization and Integrated Discovery, https://david.ncifcrf.gov/) bioinformatic online database. The cluster with the most enriched proteins exhibiting strong PPI identified from STRING analysis was uploaded as an official gene symbol in DAVID resources. The enriched pathway curated by KEGG (Kyoto Encyclopaedia of Genes and Genomes, (https://www.kegg.jp/kegg/mapper/color.html) was used to elucidate the differentially regulated protein molecular mechanisms.

### Statistical Analysis

Statistical analysis comparing the quantitative data from differential protein expression between differ-SH-SY5Y neural cells vs undifferentiated SH-SY5Y cells and 6-OHDA treated differ-SH-SY5Y neural cells vs untreated differ-SH-SY5Y cells were performed using a two-tailed Student’s *t*-test. All statistical analyses were performed with GraphPad Prism version 9.0. Differentially expressed proteins that displayed the difference in Log2 Fold change (< or > 0) with *p* < 0.05 were regarded as statistically significant.

## Results

### Label-Free Spectrometry Identification and Quantification of Differentially Regulated Proteins

**Fig. 1:**
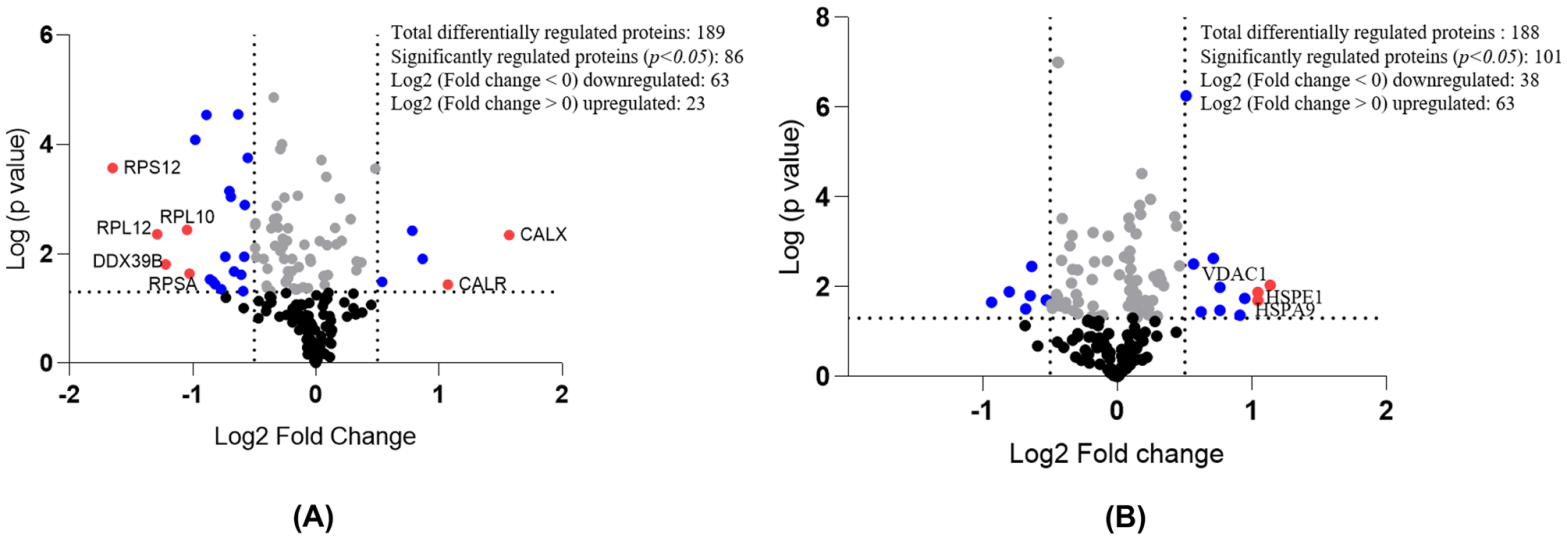
Quantitative proteomic analysis of neuronal development and 6-OHDA induced neurodegeneration on differ-SH-SY5Y neural cells. Volcano plot shows the differentially regulated protein in **(A)** Differ-SH-SY5Y neural cells **(B)** 6-OHDA induced neurodegeneration in differ-SH-SY5Y neural cells. The horizontal coordinate (x-axis) represents the difference in fold change (logarithmic transformation at the base of 2), and the vertical coordinate (Y-axis) is the significant difference of *p*-value (logarithmic transformation at the base of 10). Proteins that are onefold significantly regulated are presented as red dots, while those that had 0.5 to onefold change are shown as blue dots. Proteins with fold change less than 0.5-fold change are presented as grey dots. Black dots represent proteins that do not have a statistically significant fold-change

The protein profiling of differ-SH-SY5Y neural cells vs undifferentiated SH-SY5Y cells and 6-OHDA induced neurodegeneration vs untreated differ-SH-SY5Y were analysed using PEAKS X + software. The label-free tandem liquid mass spectrometry (LC–MS/MS) identified a total of 3261 and 3873 proteins for the differentiated and undifferentiated SH-SY5Y neural cells, respectively. Around 687 and 941 proteins were common in all the biological triplicates in differ-SH-SY5Y and undifferentiated SH-SY5Y cells, respectively. When we compared the standard protein sets between differentiated and undifferentiated SH-SY5Y neural cells, we found a total of 189 (Table 1) common proteins appeared in both protein data sets with 86 statistically significantly differentiated proteins with *p* < 0.05. Among 86 significantly regulated proteins, 63 proteins displayed a significant upregulation, while 23 proteins were downregulated. The Log_2_ fold-change distribution of differentially regulated protein revealed seven proteins exhibiting a difference of onefold change [RS12 (40S ribosomal protein S12), RL10 (60S ribosomal protein L10), RL12 (60S ribosomal protein L12), DX39B (Spliceosome RNA helicase DDX39B), RSSA (40S ribosomal protein SA), CALX (Calnexin) and CALR (Calreticulin)], 21 proteins with the 0.5 − onefold change, 60 proteins showing 0.5 − onefold-change and 101 proteins with no statistical difference fold change (Fig. 1A).

**Table 1:** Up- and downregulated proteins in differ-SH-SY5Y neural cells with average log2 fold change protein ratios between replicates

**Upregulated proteins**					
**Uniprot Accession**	**Description of protein**	**Symbol**	**Average mass**	***P* values**	**Log2 (Fold change)**
P27824	Calnexin	CANX	67,568	4.5E-03	1.571
P27797	Calreticulin	CALR	48,142	3.7E-02	1.072
P09455	Retinol-binding protein 1	RBP1	15,850	1.2E-02	0.867
P07602	Prosaposin	PSAP	58,113	3.8E-03	0.783
P30101	Protein disulfide-isomerase A3	PDIA3	56,782	3.3E-02	0.539
P30044	Peroxiredoxin-5 mitochondrial	PRDX5	22,086	2.8E-04	0.483
P07237	Protein disulfide-isomerase	P4HB	57,116	1.4E-02	0.370
Q71DI3	Histone H3.2	HIST2H3A	15,388	1.4E-02	0.333
Q12905	Interleukin enhancer-binding factor 2	ILF2	43,062	2.0E-02	0.330
P08670	Vimentin	VIM	53,652	2.3E-03	0.282
Q15084	Protein disulfide-isomerase A6	PDIA6	48,121	5.8E-03	0.209
Q06830	Peroxiredoxin-1	PRDX1	22,110	9.7E-04	0.197
P14625	Endoplasmin	HSP90B1	92,469	6.8E-03	0.167
Q99714	3-hydroxyacyl-CoA dehydrogenase type-2	HSD17B10	26,923	3.4E-03	0.156
P09429	High mobility group protein B1	HMGB1	24,894	2.5E-02	0.099
P23284	Peptidyl-prolyl cis–trans isomerase B	PPIB	23,743	7.0E-03	0.092
P61978	Heterogeneous nuclear ribonucleoprotein K	HNRNPK	50,976	3.9E-04	0.085
P07910	Heterogeneous nuclear ribonucleoproteins C1/C2	HNRNPC	33,670	3.7E-02	0.072
P0DP23	Calmodulin-1	CALM1	16,838	2.6E-02	0.066
P0DP24	Calmodulin-2	CALM2	16,838	2.6E-02	0.066
P0DP25	Calmodulin-3	CALM3	16,838	2.6E-02	0.066
Q16555	Dihydropyrimidinase-related protein 2	DPYSL2	62,294	1.9E-04	0.045
P31946	14–3-3 protein beta/alpha	YWHAB	28,082	5.8E-03	0.031

**Downregulated proteins**					
**Uniprot Accession**	**Description of protein**	**Symbol**	**Average mass**	***P*** **values**	**Log2 (Fold change)**
P25398	40S ribosomal protein S12	RPS12	14,515	2.7E-04	-1.648
P30050	60S ribosomal protein L12	RPL12	17,819	4.4E-03	-1.290
Q13838	Spliceosome RNA helicase DDX39B	DDX39B	48,991	1.6E-02	-1.218
P27635	60S ribosomal protein L10	RPL10	24,604	3.6E-03	-1.047
P08865	40S ribosomal protein SA	RPSA	32,854	2.3E-02	-1.023
Q86VP6	Cullin-associated NEDD8-dissociated protein 1	CAND1	136,375	8.3E-05	-0.980
P46776	60S ribosomal protein L27a	RPL27A	16,561	2.9E-05	-0.889
P49321	Nuclear autoantigenic sperm protein	NASP	85,238	2.9E-02	-0.860
P05388	60S acidic ribosomal protein P0	RPLP0	34,274	3.2E-02	-0.837
P46782	40S ribosomal protein S5	RPS5	22,876	3.6E-02	-0.816
P27695	DNA-(apurinic or apyrimidinic site) lyase	APEX1	35,555	4.4E-02	-0.769
P62913	60S ribosomal protein L11	RPL11	20,252	1.1E-02	-0.735
Q9UJZ1	Stomatin-like protein 2 mitochondrial	STOML2	38,534	7.1E-04	-0.701
P05387	60S acidic ribosomal protein P2	RPLP2	11,665	9.0E-04	-0.690
P60842	Eukaryotic initiation factor 4A-I	EIF4A1	46,154	2.1E-02	-0.662
P61088	Ubiquitin-conjugating enzyme E2 N	UBE2N	17,138	2.8E-05	-0.628
Q16658	Fascin	FSCN1	54,530	2.4E-02	-0.606
P30153	Serine/threonine-protein phosphatase 2A 65	PPP2R1A	65,309	4.8E-02	-0.591
	kDa regulatory subunit A alpha isoform				
P62081	40S ribosomal protein S7	RPS7	22,127	1.1E-02	-0.580
P37108	Signal recognition particle 14 kDa protein	SRP14	14,570	1.3E-03	-0.578
P62701	40S ribosomal protein S4 X isoform	RPS4X	29,598	1.8E-04	-0.552
P62906	60S ribosomal protein L10a	RPL10A	24,831	3.0E-03	-0.494
P49327	Fatty acid synthase	FASN	273,424	7.8E-03	-0.494
Q15366	Poly(rC)-binding protein 2	PCBP2	38,580	2.8E-03	-0.490
P38159	RNA-binding motif protein X chromosome	RBMX	42,332	1.2E-02	-0.484
P83731	60S ribosomal protein L24	RPL24	17,779	1.2E-02	-0.423
P35637	RNA-binding protein FUS	FUS	53,426	3.8E-02	-0.398
P61981	14–3-3 protein gamma	YWHAG	28,303	4.2E-02	-0.394
P24752	Acetyl-CoA acetyltransferase mitochondrial	ACAT1	45,200	1.9E-02	-0.392
P13639	Elongation factor 2	EEF2	95,338	4.8E-02	-0.370
P52565	Rho GDP-dissociation inhibitor 1	ARHGDIA	23,207	3.4E-03	-0.360
P15121	Aldose reductase	AKR1B1	35,853	1.4E-05	-0.340
P14174	Macrophage migration inhibitory factor	MIF	12,476	2.3E-03	-0.340
Q99525	Histone H4-like protein type G	HIST1H4G	11,009	7.1E-03	-0.329
P19338	Nucleolin	NCL	76,615	1.3E-03	-0.317
P05386	60S acidic ribosomal protein P1	RPLP1	11,514	2.3E-03	-0.315
Q14103	Heterogeneous nuclear ribonucleoprotein D0	HNRNPD	38,434	3.3E-03	-0.306
P36578	60S ribosomal protein L4	RPL4	47,697	8.9E-03	-0.288
P62851	40S ribosomal protein S25	RPS25	13,742	1.2E-04	-0.286
P23396	40S ribosomal protein S3	RPS3	26,688	7.3E-03	-0.279
P24534	Elongation factor 1-beta	EEF1B2	24,764	9.9E-05	-0.274
P07195	L-lactate dehydrogenase B chain	LDHB	36,639	7.4E-03	-0.269
P14618	Pyruvate kinase PKM	PKM	57,937	5.3E-03	-0.267
P00558	Phosphoglycerate kinase 1	PGK1	44,615	1.2E-02	-0.257
P09211	Glutathione S-transferase P	GSTP1	23,356	9.3E-04	-0.255
Q01105	Protein SET	SET	33,489	3.3E-02	-0.243
P46781	40S ribosomal protein S9	RPS9	22,591	3.1E-02	-0.240
P0C0S5	Histone H2A.Z	H2AFZ	13,553	5.8E-03	-0.234
P18124	60S ribosomal protein L7	RPL7	29,226	2.1E-02	-0.226
P50395	Rab GDP dissociation inhibitor beta	GDI2	50,663	3.4E-03	-0.226
Q02878	60S ribosomal protein L6	RPL6	32,728	2.8E-02	-0.224
P06748	Nucleophosmin	NPM1	32,575	2.1E-02	-0.196
P40227	T-complex protein 1 subunit zeta	CCT6A	58,024	1.2E-02	-0.193
P30041	Peroxiredoxin-6	PRDX6	25,035	4.3E-02	-0.180
P62258	14–3-3 protein epsilon	YWHAE	29,174	4.5E-02	-0.163
Q71UI9	Histone H2A.V	H2AFV	13,509	4.5E-02	-0.152
P11142	Heat shock cognate 71 kDa protein	HSPA8	70,898	8.7E-04	-0.146
P40926	Malate dehydrogenase mitochondrial	MDH2	35,503	1.8E-02	-0.138
P52272	Heterogeneous nuclear ribonucleoprotein M	HNRNPM	77,516	4.1E-02	-0.105
P62805	Histone H4	HIST1H4A	11,367	6.8E-03	-0.092
P10809	60 kDa heat shock protein mitochondrial	HSPD1	61,055	4.3E-02	-0.063
P06733	Alpha-enolase	ENO1	47,169	1.2E-02	-0.045
P62277	40S ribosomal protein S13	RPS13	17,222	1.4E-02	-0.041

On the other hand, the 6-OHDA treated differ-SH-SY5Y neural cells yielded 3636 proteins with 293 proteins shared the common data sets. When the protein profiles from the 6-OHDA treatment were compared with that of the untreated differentiated SH-SY5Y cells, a total of 188 proteins matched both protein data sets with a total of 101 proteins (Table 2) exhibited statistical significance difference. Among 101 significantly regulated proteins, 63 proteins were upregulated, and 38 proteins downregulated. The Log2 fold-change distribution of differentially regulated proteins displays three proteins with the difference of onefold change [VDAC1 (Voltage-dependent anion-selective channel protein 1), HSPA9 (Stress-70 protein mitochondrial), HSPE1 (10 kDa heat shock protein mitochondrial)], 15 proteins with a 0.5 − onefold-change difference, 84 proteins showing 0.5 − onefold-change and 86 proteins with no statistical difference fold-change (Fig. 1B).

**Table 2:** Up- and downregulated proteins in 6-OHDA induced neurodegeneration on differ-SH-SY5Y cells with average 6-OHDA/untreated protein ratios between replicates

**Upregulated proteins**					
**Uniprot** **Accession**	**Description of protein**	**Symbol**	**Average** **mass**	**P value**	**Log2 (Fold** **change)**
P21796	Voltage-dependent anion-selective channel protein 1	VDAC1	61546	9.20E-03	1.135
P38646	Stress-70 protein mitochondrial	HSPA9	147362	2.00E-02	1.044
P61604	10 kDa heat shock protein mitochondrial	HSPE1	21864	1.33E-02	1.044
P30049	ATP synthase subunit delta mitochondrial	ATP5F1D	34980	1.80E-02	0.945
P08758	Annexin A5	ANXA5	71874	4.29E-02	0.911
P49411	Elongation factor Tu mitochondrial	TUFM	99084	3.32E-02	0.762
P40939	Trifunctional enzyme subunit alpha mitochondrial	HADHA	166000	1.03E-02	0.761
Q9Y277	Voltage-dependent anion-selective channel protein 3	VDAC3	61318	2.31E-03	0.712
P35232	Prohibitin	PHB	59608	3.61E-02	0.622
Q99623	Prohibitin-2	PHB2	66592	3.10E-03	0.565
Q9UJZ1	Stomatin-like protein 2 mitochondrial	STOML2	77068	5.50E-07	0.511
P24752	Acetyl-CoA acetyltransferase mitochondrial	ACAT1	90400	3.38E-03	0.462
O15240	Neurosecretory protein VGF	VGF	134516	4.43E-04	0.439
P05141	ADP/ATP translocase 2	SLC25A5	65704	2.74E-04	0.425
O00571	ATP-dependent RNA helicase DDX3X	DDX3X	146488	9.43E-03	0.343
P04792	Heat shock protein beta-1	HSPB1	45566	6.85E-03	0.324
P06576	ATP synthase subunit beta mitochondrial	ATP5F1B	113120	5.29E-03	0.310
P25705	ATP synthase subunit alpha mitochondrial	ATP5F1A	119502	7.78E-03	0.302
P49327	Fatty acid synthase	FASN	546848	4.45E-02	0.296
P07355	Annexin A2	ANXA2	77208	5.93E-03	0.273
P11021	Endoplasmic reticulum chaperone BiP	HSPA5	144666	2.02E-02	0.261
P30101	Protein disulfide-isomerase A3	PDIA3	113564	1.13E-04	0.246
P45880	Voltage-dependent anion-selective channel protein 2	VDAC2	63134	3.08E-02	0.233
Q08211	ATP-dependent RNA helicase A	DHX9	281916	4.21E-02	0.230
P27824	Calnexin	CANX	135136	2.00E-02	0.209
Q9BVA1	Tubulin beta-2B chain	TUBB2B	99906	3.02E-05	0.180
Q15084	Protein disulfide-isomerase A6	PDIA6	96242	3.45E-02	0.171
P16104	Histone H2AX OS=Homo sapiens	H2AFX	30290	2.43E-04	0.171
P14625	Endoplasmin	HSP90B1	184938	1.53E-04	0.166
Q13509	Tubulin beta-3 chain	TUBB3	100866	3.50E-02	0.158
P23246	Splicing factor proline- and glutamine-rich	SFPQ	152300	2.12E-02	0.158
P62805	Histone H4	HIST1H4A	22734	4.75E-02	0.148
Q9P0M6	Core histone macro-H2A.2	H2AFY2	80116	7.24E-03	0.143
Q9BUF	Tubulin beta-6 chain	TUBB6	99714	1.45E-02	0.141
5 P07602	Prosaposin	PSAP	116226	6.53E-04	0.138
Q99714	3-hydroxyacyl-CoA dehydrogenase type-2	HSD17B10	53846	1.81E-02	0.128
P23284	Peptidyl-prolyl cis-trans isomerase B	PPIB	47486	4.24E-02	0.128
O75367	Core histone macro-H2A.1	H2AFY	79234	2.54E-02	0.127
P10809	60 kDa heat shock protein mitochondrial	HSPD1	122110	1.10E-02	0.112
P68104	Elongation factor 1-alpha 1	EEF1A1	100282	4.78E-02	0.111
P06899	Histone H2B type 1-J	HIST1H2BJ	27808	1.13E-02	0.108
P23527	Histone H2B type 1-O	HIST1H2BO	27812	1.13E-02	0.108
P33778	Histone H2B type 1-B	HIST1H2BB	27900	1.13E-02	0.108
Q16778	Histone H2B type 2-E	HIST2H2BE	27,840	1.13E-02	0.108
P62829	60S ribosomal protein L23	RPL23	29,730	1.98E-02	0.106
P27797	Calreticulin	CALR	96,284	4.01E-03	0.099
O60814	Histone H2B type 1-K	HIST1H2BK	27,780	1.19E-02	0.094
P57053	Histone H2B type F-S	H2BFS	27,888	1.19E-02	0.094
P58876	Histone H2B type 1-D	HIST1H2BD	27,872	1.19E-02	0.094
P62807	Histone H2B type 1-C/E/F/G/I	HIST1H2BC	27,812	1.19E-02	0.094
Q5QNW6	Histone H2B type 2-F	HIST2H2BF	27,840	1.19E-02	0.094
Q93079	Histone H2B type 1-H	HIST1H2BH	27,784	1.19E-02	0.094
Q99877	Histone H2B type 1-N	HIST1H2BN	27,844	1.19E-02	0.094
Q99879	Histone H2B type 1-M	HIST1H2BM	27,978	1.19E-02	0.094
P60709	Actin cytoplasmic 1	ACTB	83,474	4.58E-04	0.092
P63261	Actin cytoplasmic 2	ACTG1	83,586	1.59E-03	0.092
Q71U36	Tubulin alpha-1A chain	TUBA1A	100,272	6.86E-03	0.091
P68032	Actin alpha cardiac muscle 1	ACTC1	84,038	2.39E-03	0.087
P68363	Tubulin alpha-1B chain	TUBA1B	100,304	2.94E-04	0.085
P07437	Tubulin beta chain	TUBB	99,342	2.66E-02	0.081
Q05639	Elongation factor 1-alpha 2	EEF1A2	100,940	2.25E-03	0.069
Q00610	Clathrin heavy chain 1	CLTC	383,226	2.15E-03	0.052
P62249	40S ribosomal protein S16	RPS16	32,890	5.39E-03	0.013

**Downregulated proteins**					
**Uniprot Accession**	**Description of protein**	**Symbol**	**Average mass**	***P* value**	**Log2 (Fold Change)**
P32119	Peroxiredoxin-2	PRDX2	43,784	2.19E-02	-0.935
P55072	Transitional endoplasmic reticulum ATPase	VCP	178,644	1.29E-02	-0.803
P30041	Peroxiredoxin-6	PRDX6	50,070	3.09E-02	-0.683
P19338	Nucleolin	NCL	153,230	1.56E-02	-0.648
P36578	60S ribosomal protein L4	RPL4	95,394	3.50E-03	-0.637
P31150	Rab GDP dissociation inhibitor alpha	GDI1	101,166	1.97E-02	-0.527
P60028	Rab GDP dissociation inhibitor alpha	GDI1	101,166	1.97E-02	-0.527
P62753	40S ribosomal protein S6	RPS6	57,362	2.45E-02	-0.490
P14174	Macrophage migration inhibitory factor	MIF	24,952	3.02E-02	-0.482
P62826	GTP-binding nuclear protein Ran	RAN	48,846	1.48E-02	-0.454
P60174	Triosephosphate isomerase	TPI1	61,582	2.52E-02	-0.450
P00338	L-lactate dehydrogenase A chain	LDHA	73,378	1.66E-02	-0.446
P0DP23	Calmodulin-1	CALM1	33,676	9.96E-08	-0.442
P0DP24	Calmodulin-2	CALM2	33,676	9.96E-08	-0.442
P0DP25	Calmodulin-3	CALM3	33,676	9.96E-08	-0.442
Q16555	Dihydropyrimidinase-related protein 2	DPYSL2	124,588	2.66E-02	-0.427
P62841	40S ribosomal protein S15	RPS15	34,080	2.58E-03	-0.413
Q06830	Peroxiredoxin-1	PRDX1	44,220	3.01E-04	-0.411
P07195	L-lactate dehydrogenase B chain	LDHB	73,278	2.98E-02	-0.383
P62304	Small nuclear ribonucleoprotein E	SNRPE	21,608	2.24E-02	-0.383
Q13263	Transcription intermediary factor 1-beta	TRIM28	177,100	1.22E-03	-0.351
Q12905	Interleukin enhancer-binding factor 2	ILF2	86,124	8.24E-03	-0.342
P06733	Alpha-enolase	ENO1	94,338	7.24E-04	-0.336
P46782	40S ribosomal protein S5	RPS5	45,752	4.07E-03	-0.331
P29373	Cellular retinoic acid-binding protein 2	CRABP2	31,386	3.38E-02	-0.327
P12277	Creatine kinase B-type	CKB	85,288	1.49E-02	-0.306
P30153	Serine/threonine-protein phosphatase 2A 65 kDa	PPP2R1A	130,618	3.98E-02	-0.295
	regulatory subunit A alpha isoform				
P52565	Rho GDP-dissociation inhibitor 1	ARHGDIA	46,414	4.18E-03	-0.293
P06744	Glucose-6-phosphate isomerase	GPI	126,294	2.18E-02	-0.290
P63104	14–3-3 protein zeta/delta	YWHAZ	55,490	4.51E-02	-0.189
Q14103	Heterogeneous nuclear ribonucleoprotein D0	HNRNPD	76,868	6.85E-03	-0.188
P62851	40S ribosomal protein S25	RPS	27,484	6.19E-04	-0.180
P68431	Histone H3.1	HIST1H3A	30,808	2.69E-02	-0.180
P37802	Transgelin-2	TAGLN	44,782	2.79E-03	-0.170
P62081	40S ribosomal protein S7	RPS	44,254	4.50E-02	-0.133
P61978	Heterogeneous nuclear ribonucleoprotein K	HNRNPK	101,952	4.36E-02	-0.111
P62318	Small nuclear ribonucleoprotein Sm D3	SNRPD	27,832	2.65E-03	-0.074
P13639	Elongation factor 2	EEF	190,676	7.45E-04	-0.068

### Biocomputational Analysis of Differentially Regulated Proteins

#### Functional Annotation Analysis of Differentially Regulated Proteins Using PANTHER

**Fig. 2:**
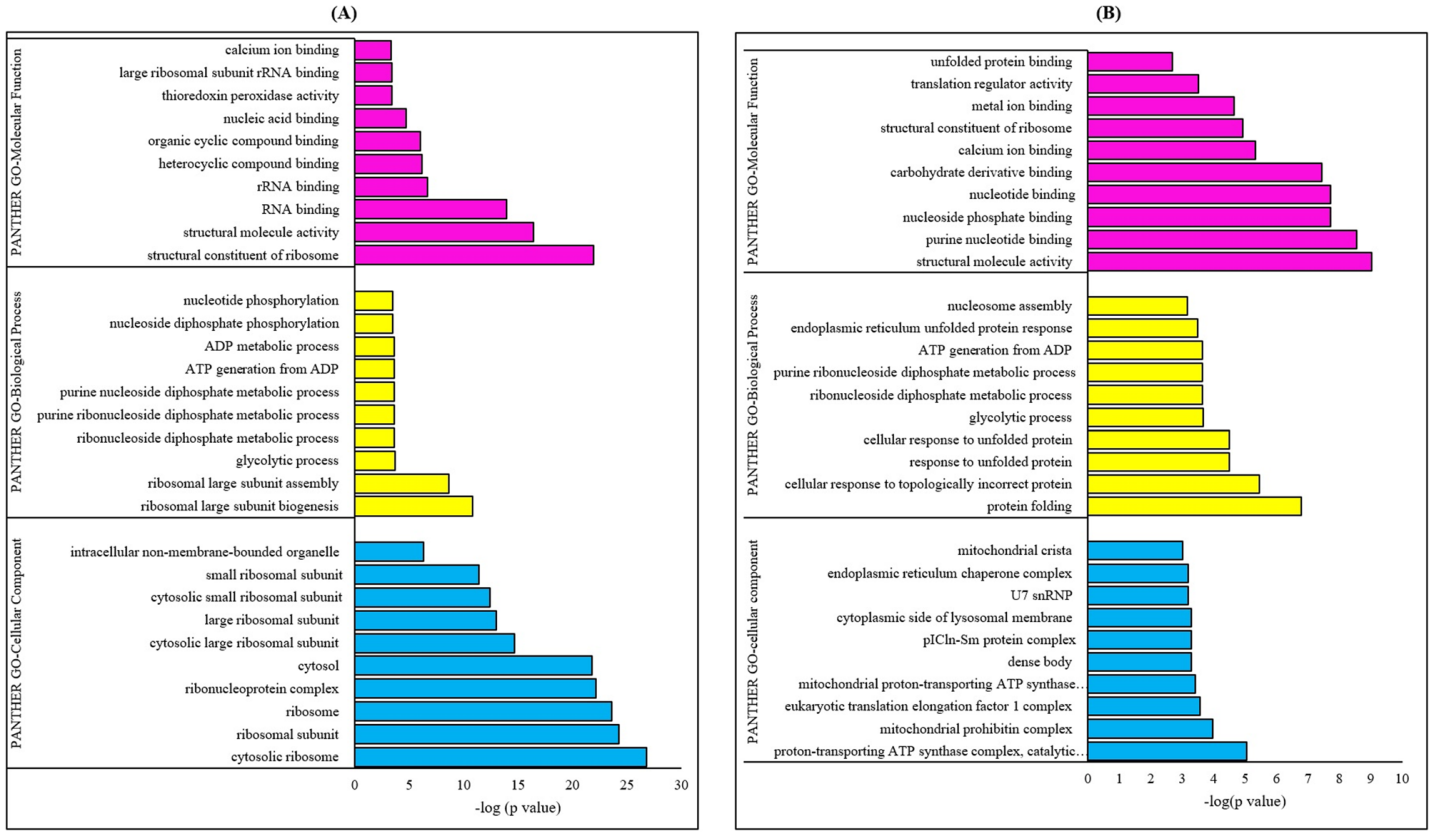
The GO classification terms of molecular function, biological process, and cellular component using PANTHER online database on differentially regulated proteins *(p* < 0.05*)* in (A) Differ-SH-SY5Y neural cells in comparison with undifferentiated SH-SY5Y neural cells and (B) 6-OHDA treated differ-SH-SY5Y neural cells in comparison with untreated differ-SH-SY5Y neural cells

To further elucidate the changes in cellular processes, the gene ontology (GO) analysis of the 86 differentially expressed proteins (*p* < 0.05) in the differ-SH-SY5Y neural cells were investigated using the PANTHER bioinformatics database. These proteins were analysed based on changes in the GO term classification clusters’ biological process, molecular function, and cellular component. The GO-molecular function of differ-SH-SY5Y neural cells showed a strong correlation to the structural modulation, activity and binding of ribosomes and ribosomal proteins. As for the GO-biological process, the differentially expressed proteins modulated the ribosomal assembly, biogenesis, glycolytic process (ATP generation) and DNA synthesis (purine nucleoside and ribonucleotide diphosphate phosphorylation and metabolic process). These molecular and biological processes were identified as concentrated at cytosolic ribosome units, as shown in the GO-cellular component (Fig. 2A).

Next, for the 6-OHDA induced neurodegeneration in differ-SH-SY5Y, the GO-molecular function showed the association of the differentially regulated proteins to the DNA structural alterations (structural molecule, purine, nucleoside, nucleotide), changes in structural constituents of ribosomes, metabolic processes (carbohydrate derivative binding), calcium cell signalling pathway (Calcium ion binding), metal ion homeostasis (metal ion binding) and unfolded protein binding. For the GO-biological process, the analysis revealed 6-OHDA induced neurodegeneration causes enrichment of the cellular mechanisms that identify and respond to misfolded/unfolded proteins (i.e. protein folding, cellular response to topologically incorrect protein, cellular response to unfolded proteins, endoplasmic reticulum unfolded protein response), metabolic activity (glycolytic activity, ATP generation), DNA structural changes (purine ribonucleoside diphosphate metabolic process), and apoptotic nucleosome assembly. The proteins localized in GO-cellular components were identified to be mitochondria (i.e. proton-transporting ATP synthase complex, mitochondrial prohibitin complex, mitochondria cristae), neuronal synapse or ribbon synapse (dense body), ribosome (i.e. eukaryotic translation elongation factor 1 complex, cytosolic RNA splicing (pIC1n-Sn protein complex, U7 snRNP) and endoplasmic reticulum (Fig. 2B).

## STRING Protein–Protein Interaction (PPI) Network Analysis 

**Fig. 3:**
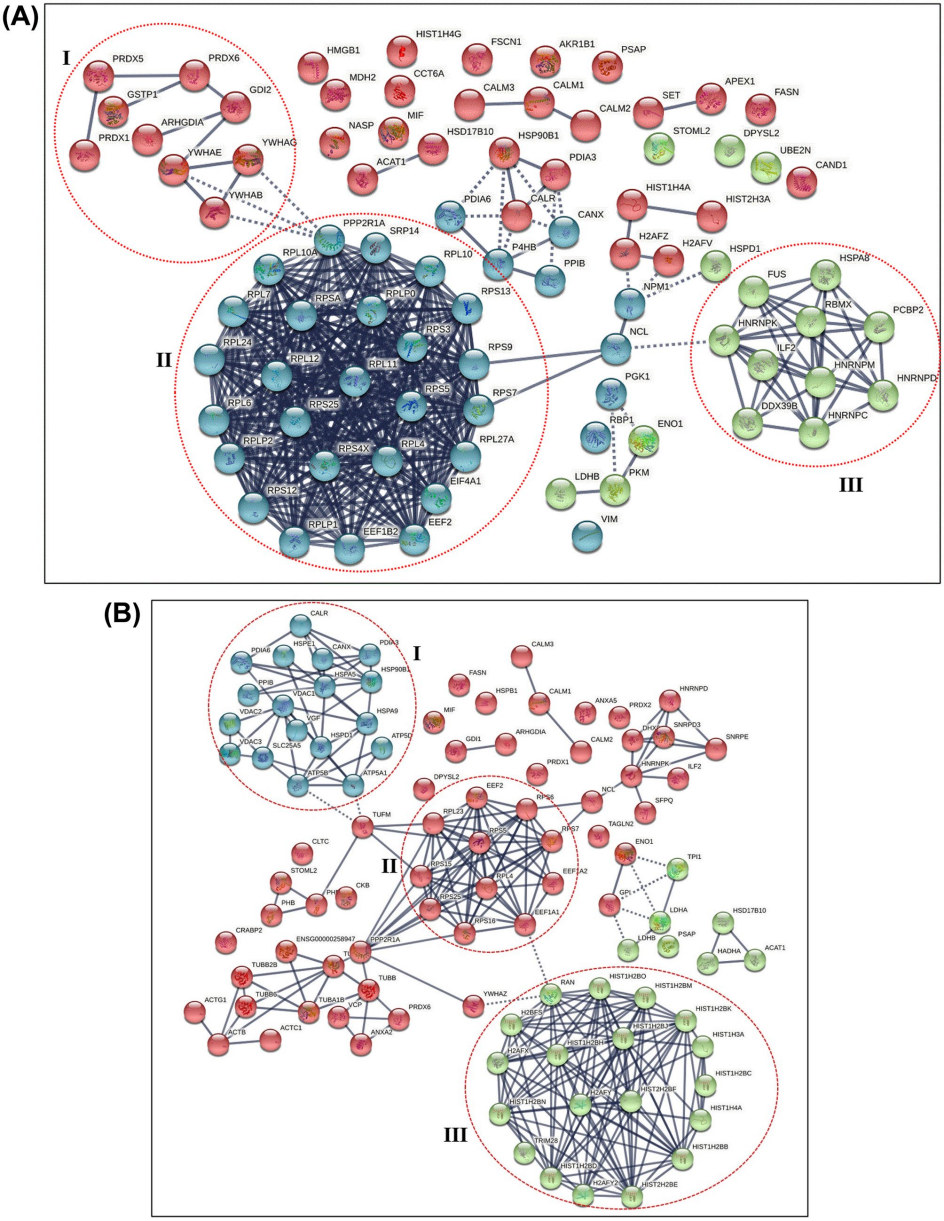
(A) The STRING PPI analysis of differentially regulated proteins in differ-SH-SY5Y neural cells vs undifferentiated SH-SY5Y cells. (B) STRING Protein–protein interaction network of 6-OHDA induced neurodegeneration on differ-SH-SY5Y neural cells. The STRING PPI analysis of differentially regulated proteins in 6-OHDA treatment on differ-SH-SY5Y neural cells vs untreated differ-SH-SY5Y cells. The red dotted circles show protein clusters with the highest confidence (0.9) interactions. The edges were drawn with different line thickness that indicates the strength of data support

The STRING PPI network in differ-SH-SY5Y neural cells generated a total of 396 edges and 86 nodes with a PPI enrichment *p*-value of < *1.00* × *10*^*–16*^, which was generated using the highest confidence (0.9) category (Fig. 3A). The PPI enrichment clusters showed a local clustering coefficient of 0.658. A total of three clusters were generated using K-means clustering. Proteins in **cluster I** include peroxiredoxin-6 (PRDX6), peroxiredoxin-5 mitochondrial (PRDX5), rho GDP-dissociation inhibitor 1 (ARHGDIA), peroxiredoxin-1 (PRDX1), 14–3-3 protein epsilon (YWHAE), 14–3-3 protein beta/alpha (YWHAB), 14–3-3 protein gamma (YWHAG), Rab GDP dissociation inhibitor beta (GDI2) and Glutathione S-transferase P (GSTP1), whilst proteins in **cluster II** comprises of EEF1B2 (Elongation factor 1-beta), EEF2 (Elongation factor 2), EIF4A1 (Eukaryotic initiation factor 4A-I), PPP2RIA (Serine/threonine-protein phosphatase 2A 65 kDa regulatory subunit A alpha isoform), RPL10 (60S ribosomal protein L10), RPL10A (60S ribosomal protein L10a), RPL11 (60S ribosomal protein L11), RPL12 (60S ribosomal protein L12), RPL24 (Ribosomal protein L24), RPL27A (Ribosomal protein L27a), RPL4 (L ribosomal proteins), RPL6 (60S ribosomal protein L6), RPL7 (60S ribosomal protein L7), RPLP0 (60S acidic ribosomal protein P0), RPLP1 (60S acidic ribosomal protein P1), RPLP2 (60S acidic ribosomal protein P2), RPS12 (Ribosomal protein S12), RPS13 (Ribosomal protein S13), RPS25 (Ribosomal protein S25), RPS3 (40S ribosomal protein S3), RPS4X (Ribosomal protein S4), RPS5 (Ribosomal protein S5), RPS7 (40S ribosomal protein S7), RPS9 (Ribosomal protein S9), RPSA (40S ribosomal protein SA) and SRP14 (Signal recognition particle 14 kDa protein). **Cluster III** contains proteins such as DDX39B (Spliceosome RNA helicase DDX39B), FUS (RNA-binding protein FUS), HNRNPC (Heterogeneous nuclear ribonucleoproteins C1/C2), HNRNPD (Heterogeneous nuclear ribonucleoprotein D0), HNRNPK (Heterogeneous nuclear ribonucleoprotein K), HNRNPM (Heterogeneous nuclear ribonucleoprotein M), HSPA8 (Heat shock cognate 71 kDa protein), ILF2 (Interleukin enhancer-binding factor 2), RBMX (RNA-binding motif protein) and PCBP2 (Poly(rC)-binding protein 2) (Fig. 3A). As cluster II exhibits the highest confidence edges with strong populated PPI, proteins in this cluster were further analysed for the pathway enrichment using the DAVID TOOL and KEGG databases.

The STRING PPI networks of the 6-OHDA induced neurodegeneration in differ-SH-SY5Y neural cells that generated a total of 245 edges and 97 nodes with PPI enrichment *p*-value < *1.0* × *10*^*–16*^ is shown in Fig. 3B. The networks were derived using the highest confidence (0.9) category that demonstrates three distinct clusters generated using K-means clustering. The PPI enrichment clusters exhibited a local clustering coefficient of 0.676 that generated three distinct clusters using K-means clustering. Proteins in **cluster I** include CALR (calreticulin), CANX (calnexin), HSP90B1 (endoplasmin), HSPA5 (78 kDa glucose-regulated protein), HSPA9 (stress-70 protein, mitochondrial), HSPD1 (60 kDa heat shock protein), HSPE1 (10 kDa heat shock protein), PDIA3 (protein disulfide-isomerase A3), PDIA6 (protein disulfide-isomerase A6), PPIB (peptidyl-prolyl cis–trans isomerase B), SLC25A5 (ADP/ATP translocase 2), VDAC1 (voltage-dependent anion-selective channel protein 1), VDAC2 (voltage-dependent anion-selective channel protein 2), VDAC3 (voltage-dependent anion-selective channel protein 3), ATP5A1 (ATP synthase subunit alpha, mitochondrial), ATP5B (ATP synthase subunit beta, mitochondrial) ATP5D (ATP synthase subunit delta, mitochondrial) and VGF (neurosecretory protein VGF). **Cluster II** includes RPL4 (L ribosomal proteins), RPS16 (Ribosomal protein S16), RPS6 (40S ribosomal protein S6), RPS15 (Ribosomal protein S15), RPS25 (40S ribosomal protein S25), RPS16 (40S ribosomal protein S16), RPL23 (60S ribosomal protein L23), EEF1A1 (Elongation factor 1-alpha 1), EEF2 (Elongation factor 2), RPS7 (40S ribosomal protein S7), EEF1A2 (Elongation factor 1-alpha 2), whilst proteins in **cluster III** include histone proteins such as H2AFX (Histone H2AX), H2AFY (Core histone macro-H2A.1), H2AFY2 (Core histone macro-H2A.2), H2BFS (Histone H2B type F-S), HIST1H2BB (Histone H2B type 1-B), HIST1H2BC (Histone H2B type 1-C), HIST1H2BD (Histone H2B type 1-D), HIST1H2BH (Histone H2B type 1-H), HIST1H2BJ (Histone H2B type 1-J), HIST1H2BK (Histone H2B type 1-K), HIST1H2BM (Histone H2B type 1-M), HIST1H2BN (Histone H2B type 1-N), HIST1H2BO (Histone H2B type 1-O), HIST1H3A (Histone cluster 1 H3 family member a), HIST1H4A (Histone cluster 1 H4 family member a), HIST2H2BE (Histone H2B type 2-E), HIST2H2BF (Histone H2B type 2-F), RAN (GTP-binding nuclear protein Ran) and TRIM28 (Transcription intermediary factor 1-beta). The strong PPI edges and highest number of protein recruitment were noted in cluster III compared to other clusters; hence proteins populated in this cluster were subjected to pathway enrichment analysis.

## Discussion

The SH-SY5Y human neuroblastoma cell line is regarded as versatile neuronal cells that can be applied in multitude neuroscience research. However, it is imperative to differentiate the highly proliferative neuroblastoma cells that entails a number matured neuron characteristics such as extension of neurite projections, increased electrical potential, expression of neuron-specific markers and neurotransmitters (Kovalevich and Langford 2013). In this regard, several studies have highlighted that RA enhances the expression of dopaminergic (Lopes et al. 2017), adrenergic and cholinergic characteristics (Hashemi et al. 2003; HES et al. 2018) in human SH-SY5Y neuroblastoma cells. On that note, our research team has previously reported that the SH-SY5Y neuroblastoma cells differentiated in RA containing low serum medium for 6 days significantly enhanced the dopamine and α-synuclein levels besides upregulating the dopamine receptor D2 (DRD2) gene expression (Magalingam et al. 2020). In continuation of our previous study, this study is aimed to gain further insights into the differential proteome expression pattern implicated by RA in human neuroblastoma cells. Next, we explored the biological and molecular pathway induced by 6-OHDA in differ-SH-SY5Y, that is known as cellular model of PD.

Our findings suggest that the RA induced differentiation of SH-SY5Y cells exhibited a significant increase in the expression of two crucial proteins, calnexin and calreticulin proteins (Fig. 1A). Calreticulin, an essential Ca^2+^ binding chaperone in the endoplasmic reticulum, is a vital entity of the calreticulin/calnexin cycle (Xiao et al. 2017). Nevertheless, the calreticulin/calnexin cycle plays a vital role in folding newly synthesized proteins for cellular differentiation or organ development (Dudek and Michalak 2013). Emerging studies have shown that calreticulin mediated suppression of oncogenic N-MYC (MYCN) resulted in increased neurite length and differentiation marker GAP-43 (Lee et al. 2019). A study on the calreticulin knock-out mouse model displayed embryonic lethality with remarkable defects in the heart, brain and body wall, suggesting the pivotal role of calreticulin in the development of the nervous system (Rauch et al. 2000). On the other hand, calnexin's role has been associated with nerve cells' myelination, as being deficient in the calnexin gene leads to myelinopathy (Kraus et al. 2010). Hence, the expression of these proteins in differ-SH-SY5Y neural cells signifies the activation of neuronal features such as myelination and suppression of oncogenic characteristics of neuroblastoma cells.

**Fig. 4:**
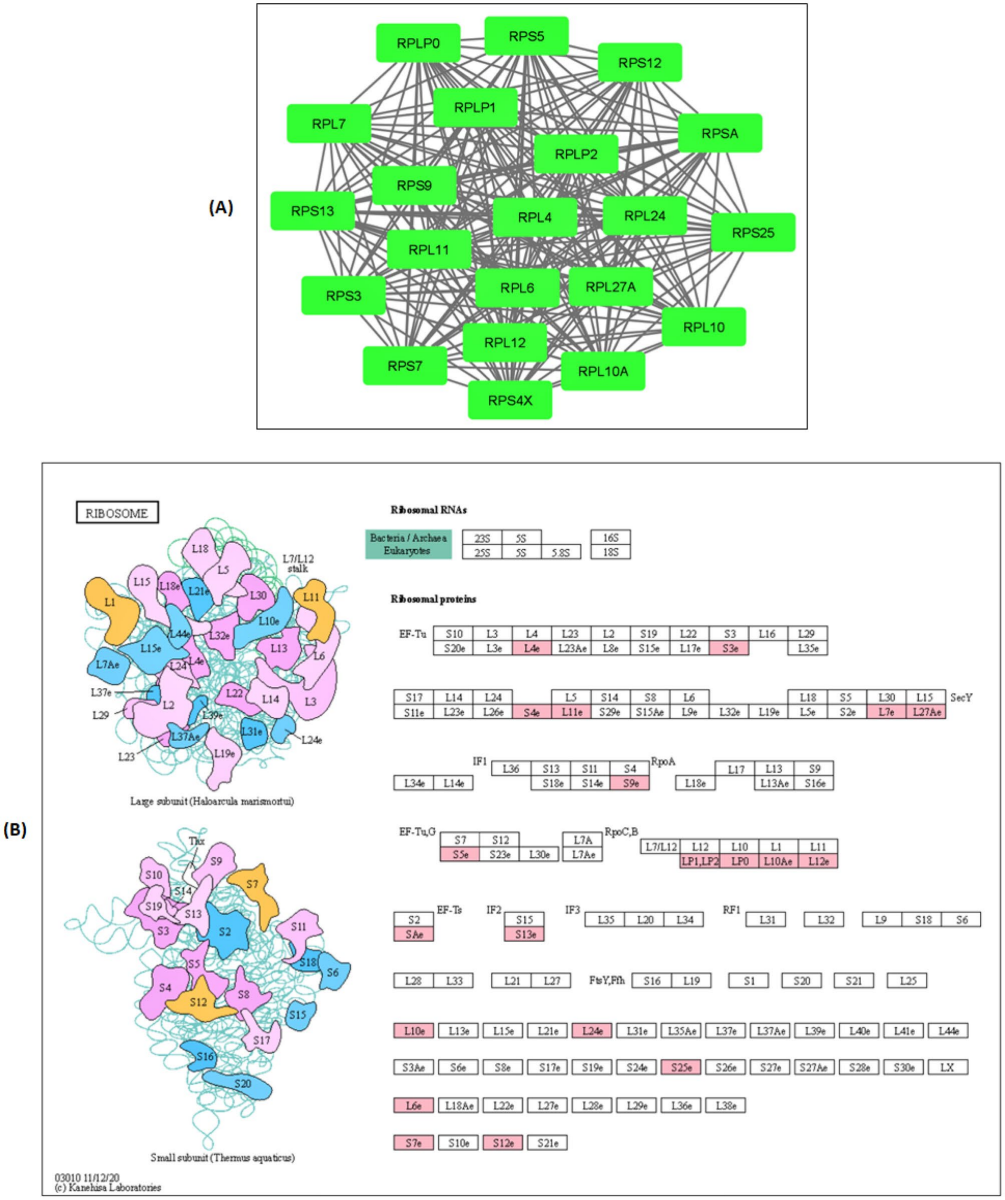
KEGG Pathway enrichment analysis of Cluster II proteins. (A) The differentially regulated cluster II protein network is visualized using Cytoscape 3.8.0. The green nodes indicate the significantly downregulated cluster II proteins (*p* < 0.05). (B) The position of cluster II proteins in the KEGG-ribosome pathway during the neuronal maturation in differ-SH-SY5Y neural cells are shown as pink-coloured boxes. The cluster II proteins involved in this pathway are ribosomal protein family members that can be classified as large subunit ribosomal proteins (RPL10, RPL10A, RPL11, RPL12, RPL24, RPL27A, RPL4, RPL6, RPL7, RPLP0, RPLP1, RPLP2) and small subunit ribosomal proteins (RPS12, RPS13 RPS25, RPS3, RPS4X, RPS5, RPS7, RPS9, RPSA) ( *Adapted from KEGG Ribosome pathway- ko03010*)

The differ-SH-SY5Y neural cells also demonstrated a significant onefold downregulation of DDX39B and ribosomal proteins, RPS12, RPL10, RPL12 and RPSA. The DDX39B is a member of the DEAD-box family of RNA helicases that involve in pre-mRNA splicing and mRNA export to the cytoplasm (Zhang et al. 2018). Intrinsically, the DDX39B promotes the unwinding of the U4/U6 snRNA duplex, which in-turn permits the binding of the U2 snRNP to the pre-mRNA in a series of ATP-dependent pre-mRNA splicing process (Shen et al. 2008). Consequently, DDX39B mediates the nuclear transport of mRNA by facilitating the interaction between THO complex and CIP29 and Aly (nuclear factors) (Folco et al. 2012). The interaction between the DDX39B with export proteins CIP29 and Aly during the formation of the conserved TREX mRNA export complex is controlled by ATP hydrolysis Dufu et al. (2010). A recent study has reported that the increased pre-ribosomal RNA levels of DDX39B augment global translation and cell proliferation of diverse cancer types. This study also showed that DDX39B knockdown cells displayed a significantly reduced stability of pre-ribosomal 47S RNA, whereas the 47S rRNA stability was unaltered in DDX39B overexpressed cells (Awasthi et al. 2018). The present study's findings suggest that the downregulation of DDX39B in differ-SH-SY5Y neural cells is closely linked to the suppression of ribosomal proteins and RNA biogenesis. The inhibition of cell proliferation in differentiated neural cells was caused by the suppression of ribosomal proteins as this event is pertinent in protein biosynthesis and cell growth. Evidence supporting this finding comes from a study by Linstrom et al*.* that suggested that silencing of ribosomal protein S9 (RPS9) elicits cell proliferation restriction mediated by the p53 tumour suppressor pathway in cancer cells. This study also pointed out that the suppression of ribosomal proteins effectively promotes differentiation processes, senescence, or apoptosis in rapidly propagating cancer cells (Lindström and Nistér 2010). According to our STRING PPI analysis, a total of 28 differentially regulated proteins were found to form the most prominent network in cluster II. Among these, 21 ribosomal proteins displayed a significant downregulation in differ-SH-SY5Y neural cells compared to undifferentiated SH-SY5Y cells (Fig. 3A). These 21 ribosomal proteins were identified to have a pivotal role in the ribosome bio-pathway as curated by the KEGG database (Fig. 4).

The ribosome is the cellular translational machinery’s principal component that decodes messenger RNA (mRNA) to produce an amino acid chain through a complementary anticodon sequence (Guimarães 2017). The manufacture of this machinery component is known as ribosome biogenesis and occurs in the nucleolus via initiation by the RNA-polymerase-1 (RNA-pol1) that mediates the transcription of rRNA genes (Drygin et al. 2010). Three RNA polymerase enzymes, RNA-pol1, RNA-pol2, RNA-pol3 and non-ribosomal factors, were responsible for generating the 90S pre-ribosomes in the nucleolus undergoes several modifications before splitting into pre-60S and pre-40S particles (Torreira et al. 2017). These particles mature into the large (60S) and small (40S) subunits for protein synthesis during the transportation from nucleolus into cytoplasm compartments through dissociation from most of the non-ribosomal factors (Delavoie et al. 2019). Emerging studies have revealed that ribosome biogenesis and protein translations are exceptionally synchronized with cellular processes, including cell division, differentiation and growth (Zhou et al. 2015). Disruption in any of these two dynamic processes could impede the crucial cellular biological processes and well-being.

Furthermore, recent evidence has documented that downregulation of the ribosomal proteins (RPs) and translation efficiency during the differentiation process contribute to reduced cell growth (Marcon et al. 2017) (Bevort and Leffers 2000) (Hayashi et al. 2014). Bevort and Leffers have reported that 31 out of 32 RPs analysed were significantly suppressed in neuronal differentiation in human NTERA2 cells induced by retinoic acid. The same study also demonstrated that the reduction in RP mRNA expression was well correlated with inhibition of the proliferation marker known as proliferating cell nuclear antigen (PCNA) (Bevort and Leffers 2000). An incredible wealth of information was contributed by Hayashi and team elucidating the association between downregulation of rRNA transcription and cellular differentiation (Hayashi et al. 2014). According to this report, the rRNA transcription was deliberately downregulated using actinomycin D, a siRNA for Pol 1-specific transcription factor IA (TIF-IA) in HL-60 and THP-1 cells differentiation potential. The attenuation of rRNA transcription was shown to enhance the cell differentiation in both cell lines and increase the differentiation marker, CD11b. They also evaluated if cell differentiation was triggered by inhibition of the cell cycle since rRNA transcription is tightly paired with cell growth. The outcome of this study showed that cell cycle arrest that occurred without affecting rRNA transcription did not stimulate differentiation in mouse hematopoietic stem cells (Hayashi et al. 2014). This data is in line with our findings that showed a significant downregulation of RPs in differ-SH-SY5Y cells indicate the augmentation in the differentiation mechanism and suppression of cell growth compared to undifferentiated SH-SY5Y neuroblastoma cells.

The 6-OHDA induced neurodegeneration model is a well-acknowledged “simulation” conceived by the PD research community to study the cellular and molecular processes, both in vitro or in vivo settings (Xicoy et al. 2017). Although the model does not demonstrate the classical pathological hallmark of PD, which is the accumulation of α-synuclein and Lewy bodies, it expresses the cardinal processes in PD, including mitochondrial dysfunction, apoptosis, ROS induced oxidative stress, neuro-inflammation, lipid peroxidation and disruption in endogenous antioxidant enzymes (Dias et al. 2013). In our study, the 6-OHDA exposure on the differ-SH-SY5Y neural cells yielded a total of 101 differentially regulated proteins. It is important to highlight that none of these proteins exhibit close association to dopaminergic, adrenergic or cholinergic neuron receptors. The exposure of 6-OHDA on differ-SH-SY5Y neural cells revealed a remarkable onefold overexpression of VDAC1, HSPE1 and HSPA9 proteins (Fig. 1B).

The VDAC1 is found abundantly on the outer membrane of mitochondria and functions as a gatekeeper for the passage of ions (Ca^2+^, K^+^, Na^+^) and metabolite substrates ATP, ADP and Pi (Camara et al. 2017). The conformational states of VDAC1 are voltage-dependent and ion selectivity, exhibiting a preference to metabolite anions in high voltage conductance (open state) and cations in low voltage conductance (closed state) (Rostovtseva and Colombini 1997). The VDAC remains in high conductance or open state during mitochondria depolarization potential in the voltage range of about -40 to + 40 mV (Hodge and Colombini 1997). The VDAC function is associated with NADH's oxidation, hence playing a pivotal role in mitochondria-mediated apoptotic signalling via interaction with pro-and anti-apoptotic mediators (Shoshan-Barmatz et al. 2017). Previous studies have reported that the overexpression of VDAC1 in cells undergoing apoptosis was mediated by increased cytosolic Ca^2+^ level. The treatment of pro-apoptosis inducer, hydrogen peroxide on Hela (human cervical adenocarcinoma) cells resulted in increased cytosolic Ca^2+^ and overexpressed oligomerised VDAC1 mediating the release of cytochrome c and apoptosis (Shoshan-Barmatz et al. 2017). Another study has further proven that a binding partner known as tubulin polymerization-promoting protein family member-3 (TPP3) promoted the oligomerisation of VDAC1 in palmitic acid-induced apoptosis of endothelial cells (Liu et al. 2020). Hence, the upregulation of VDAC1 protein expression in our finding indicates that the 6-OHDA treated differ-SH-SY5Y neural cells underwent rigorous oxidative stress-induced apoptosis, which is an expected outcome in a PD disease model.

The heat shock proteins (HSPs) are the most conserved 10 kDa evolutionary proteins known as stress-inducible proteins. The HSPE1 (HSP10) is found abundantly in mitochondria, whereas HSPA9 (HSP70) exists in the cytoplasm and nucleus. Generally, HSPs function as cytoprotective proteins with related co-chaperones under oxidative stress induced apoptosis through the initiation of repair mechanism and refolding of misfolded peptides, possible proteolysis of irreparable proteins, signalling transduction and translocation (Sharma et al. 2012). Principally, the HSPA9 assists in the transportation of nuclear-encoded proteins to the mitochondria and these proteins are subsequently refolded by HSPD1(HSP60) and its co-chaperone, HSPE1 (Voos 2013). The HSPD1-HSPE1 complex chaperonin consists of two rings arranged to conform to a barrel-shaped structure with a central cavity. This complex receives the unfolded or misfolded proteins into its central cavity and facilitates the folding process divided into three steps. In the first step, the HSPD1-HSPE1 complex binds firmly with the unfolded or misfolded proteins; then the proteins get trapped in the central cavity capped by HSPE1 refolding of the protein takes place. Finally, the correctly folded proteins are ejected from the chaperonin complex. The unfolded or partially folded proteins are redirected to the chaperonin to repeat the entire cycle until the correctly folded proteins are achieved (Jia et al. 2011). While the HSPD1 and HSPE1 are crucial housekeeping proteins for efficient mitochondria function and biogenesis, the HSPA9 is best known for its cytosolic chaperone activity in assisting protein folding, degradation and translocation. Overwhelming evidence has pointed out that HSPs have been identified to be involved in multiple pathways in promoting the anti-apoptosis effect (Ikwegbue et al. 2018). According to Li et al. the chaperone activity of HSPA9 is critical for the inhibition of caspase activation at a reaction point between the cytochrome c release and caspase-3 activation (Li et al. 2000). The overexpression of HSPA9 was also reported to indirectly inhibit the stress-induced apoptosis by preventing the conformational change and translocation of Bax into mitochondria, indicating the suppression of apoptosis through Bax inactivation and inhibition of caspase activation (Stankiewicz et al. 2005). A recent study has clarified that overexpression of HSPA9 prevents inflammation in a rat model of intracerebral haemorrhage through inhibition of inflammatory cytokines such as TNF-α, IL-β, Bax, and increased Bcl-2 levels (Lv et al. 2017). Hence, we postulate that the overexpression of HSPE1 and HSPA9 in our study is closely connected to the cytoprotective mechanism of the neuronal cells to inhibit the avalanche of apoptotic mediators released in response to 6-OHDA treatment. The HSP expression in the PD cell model is an important biomarker that paves the way for future studies in developing potential therapies targeting the HSPE1 and HSPA9 to mitigate apoptosis-induced cell death.

**Fig. 5:**
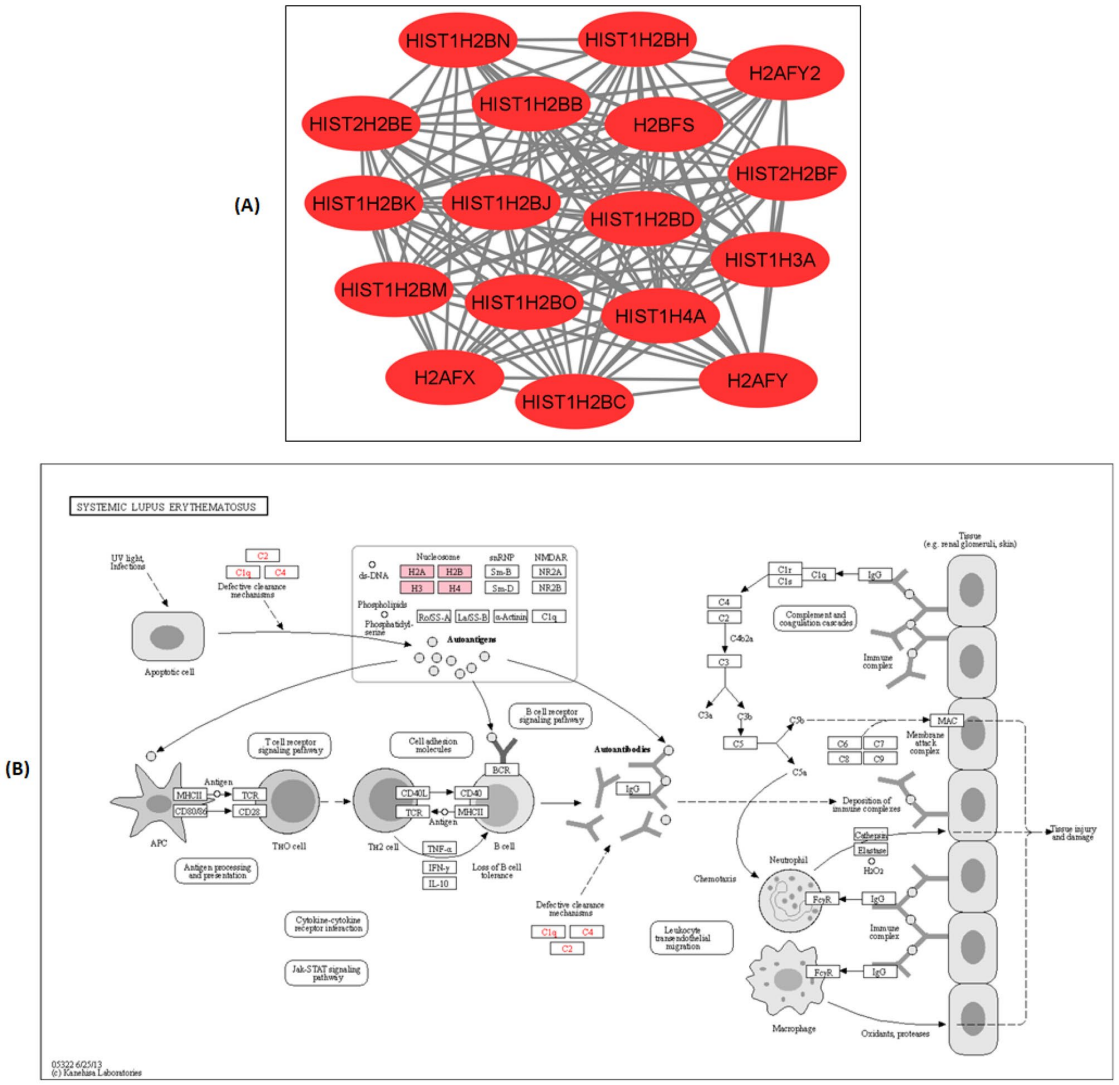
KEGG pathway enrichment analysis of cluster III proteins. (**A**) The differentially regulated cluster III protein network in differ-SH-SY5Y neural cells in response to 6-OHDA is visualized using Cytoscape 3.8.0. The Red nodes indicate the significantly upregulated cluster III proteins (*p* < 0.05). **(B)** The position of cluster III proteins in the KEGG SLE pathway during the 6-OHDA induced neurodegeneration process are shown as pink-coloured boxes. The 17 cluster III proteins involved in this pathway are from H2A histone family member (H2AFX, H2AFY, H2AFY2), Histone H2B type (H2BFS), Histone cluster 1 H2B family (HIST1H2BB, HIST1H2BC, HIST1H2BD, HIST1H2BH, HIST1H2BJ, HIST1H2BK, HIST1H2BM, HIST1H2BN, HIST1H2BO), Histone cluster 1 H3 family (HIST1H3A), Histone cluster 1 H4 family (HIST1H4A) and Histone cluster 2 H2B family (HIST2H2BE, HIST2H2BF) ( *Adapted from KEGG Systemic Lupus Erythematosus pathway- hsa05322*)

The treatment of 6-OHDA-induced neurodegeneration on differ-SH-SY5Y neural cells resulted in an enrichment of 4 clusters or pathways in STRING PPI analysis (Fig. 3B). The crosstalk between proteins populated in cluster IV of STRING PPI displaying the most prominent interaction was further investigated using KEGG pathway enrichment analysis. The KEGG bioinformatic pathway enrichment database predicted the systemic lupus erythematosus (SLE) pathway with the involvement of 17 out of 19 upregulated proteins (Fig. 5). SLE is an autoimmune disease of unknown aetiology that primarily affects women in the childbearing age (Pieterse and van der Vlag 2014). The disease is characterized by disturbances of the immune system that arise when the immune cells respond to self-antigens, mainly nuclear constituents, i.e. histones, ribonucleoproteins and DNA (Pradhan et al. 2010). For over two decades, apoptosis has been regarded as a significant source of autoantigens in SLE. Apoptosis can be actively triggered by ligation of cell surface receptors, including Fas and tumour necrosis factor receptor (TNFR) or passively via deficient in crucial cell survival signals (De Wilde et al. 2001). Apoptotic cells undergo an orderly process of morphological alterations, such as nuclear chromatin condensation, nuclear splitting, cytoskeletal disruption, cell shrinkage and membrane blebbing (Saraste and Pulkki 2000). Persistent exposure of the immune system to apoptotic bodies leads to the formation of an anti-chromatin/chromatin complex, triggering an array of inflammation in multiple organs in SLE patients. Previous studies have shown that autoantibodies' increased reactivity against histone proteins, namely histone H4 and H2B peptides, correlated with SLE disease activity (Dieker et al. 2016). Whereas in PD, the selective vulnerability of dopamine neurons to environmental toxins, intracellular accumulations of highly oxidative free radical and toxic accumulation of misfolded proteins leads to neurodegeneration (apoptosis) and activation of neuro-inflammation pathway (Hald and Lotharius 2005). As such, the neuro-inflammation pathway is triggered through activation of microglia or the “immune cell of the brain”, which releases the pro-inflammatory mediators including tumour necrosis factor (TNF-α), interleukin (IL-1β), IL-2, IL-4, interferon (IFN-γ), and nitric oxide (NO) (Liu et al. 2019). The pro-inflammatory mediators instigate further detrimental effects on nigral neurons by causing direct toxicity in neurons and induce ongoing neuro-inflammation through microglial NO activation (Jung et al. 2019). Emerging studies have reported that chronic SLE patients presented with parkinsonian syndromes, including slowness in movement, adiadochokinesia, postural rigidity, and tremor (Fabiani et al. 2002).

In our study, the histone proteins were shown to be exceptionally overexpressed in 6-OHDA induced neurodegeneration on differ-SH-SY5Y neural cells (PD cell model). Structurally, histone proteins exist as an octamer with two copies of each of the four core histone proteins H2A, H2B, H3, and H4 and two linker histones, H1 and H5 (Andrews and Luger 2011). The 147 bp of DNA packages the octamer histones to form a nucleosome. The four core histones form a conserved central motif domain known as histone fold composed of a long central α-helix structure with a short helix flanked on either side (Jiang and Pugh 2009). In comparison, the linker histone associates with nucleosome to transform nucleosomes into various high-order chromatin structures. Ideally, the histone proteins function in constructing the nucleus and fine-tuning gene expression for physiological and pathological processes (Martire and Banaszynski 2020). An increasing body of evidence suggests that besides regulating nucleosome dynamics, histone proteins are released in the extracellular field by cells undergoing apoptosis (Silk et al. 2017).

It is well established that chromatin condensation coupled with DNA fragmentation is important nuclear events during apoptosis (He et al. 2009). In response to apoptotic signals, the core and linker histones are detached from genomic DNA and released into cytoplasmic and extracellular regions. Wu et al. have reported that the timing of histone release from genomic DNA directly correlated with the advancement of the apoptosis process (Wu et al. 2002). The release of histone proteins and DNA-bound nucleosomes into the intracellular space by damaged DNA is an essential indicator of apoptosis-activated pro-inflammatory cascades (Silk et al. 2017). Once in the cytosol, histones act as a member of the damage-associated molecular pattern molecules (DAMPs), activating immune response and causing additional cytoproliferative effects (Xu et al. 2009). Along these lines, studies have revealed that core histones such as H2A, H2B, H3, H4 and linker histone H1 are frequently detected in neurons (Mishra et al. 2010), microglia (Klein et al. 2014) and macrophages (Brix et al. 1998) in response to oxidative stress (Hu et al. 2018). Notably, the degree of circulating histone and nucleosomes are elevated in cancer, infection and inflammation proposing histone as an essential biomarker in human diseases (Chen et al. 2014). Hence, the upregulation of a set of histone proteins in 6-OHDA induced neuronal death in our study is a clear indication of chromatin condensation and modification during DNA fragmentation in the apoptosis process.

In conclusion, the PD cell model is an essential in vitro platform for investigating the structural and molecular changes manifested in disease process and paving the way for future discoveries of potential therapeutic drugs that reverse these mechanisms. Ideally, a disease model should express the biomarkers that indicate the progression of PD's pathological conditions such as mitochondrial dysfunction, DNA fragmentation, neurodegeneration, and neuro-inflammation. The differ-SH-SY5Y neural cells developed from SH-SY5Y human neuroblastoma cells expressed substantial dopaminergic markers and, upon perturbation with 6-OHDA, displayed pathological changes frequently detected in PD. The differentially regulated proteins were analysed using GO functional annotation, STRING PPI and KEGG pathway enrichment databases to understand the cellular machinery’s alterations during the disease process. We suggest that the downregulation of the ribosome pathway mediates the differentiation of SH-SY5Y neuroblastoma cells and nucleosomal degradation demonstrated by upregulation of histone products in SLE pathway is a key event in 6-OHDA induced neurodegenerative process. 

## Supplementary Information

Below is the link to the electronic supplementary material.

Supplementary file1 (PDF 133 KB)
