# Supplementary material for: 6-Hydroxydopamine Induces Neurodegeneration in Terminally Differentiated SH-SY5Y Neuroblastoma Cells via Enrichment of the Nucleosomal Degradation Pathway: a Global Proteomics Approach
Source: J Mol Neurosci. 2022 Mar 8;72(5):1026–46. doi: 10.1007/s12031-021-01962-z (PMC9064865; doi:10.1007/s12031-021-01962-z)
Supplement: Supplementary file 1 — Supplementary file1 (PDF 133 KB) [file 12031_2021_1962_MOESM1_ESM.pdf]

KEGG Database Project
Kanehisa Laboratories
www.kanehisa.jp

Ref: 210234

Permission is granted to Scientific Reports of Springer Nature Ltd to publish both in print and digital under the CC BY 4.0 open access license the following KEGG pathway map images in the article "6-hydroxydopamine induce neurodegeneration in terminally differentiated SH-SY5Y neuroblastoma cells via nucleosomal degradation: A quantitative proteomic approach" written by Ammu Radhakrishnan and colleagues:

- Systemic lupus erythematosus - Homo sapiens (human) (hsa05322)
- Ribosome (ko03010)

subject to the condition that the original source is acknowledged by citing at least one KEGG paper.

Permission granted: Junko Takigawa

Junko Takigawa, Kanehisa Laboratories

Date: 8 March 2021

Copyright holder: Kanehisa Laboratories
